# A potential cause of adolescent onset Dyke-Davidoff-Masson syndrome: A case report

**DOI:** 10.1097/MD.0000000000018075

**Published:** 2019-12-20

**Authors:** Yiyang Li, Tao Zhang, Bo Li, Jing Li, Li Wang, Zhibin Jiang

**Affiliations:** a Department of Cardiology, The National Hospital of Guangxi Province; b Department of Neurology, The 923th Hospital of People's Liberation Army, Nanning, China.

**Keywords:** adolescent, Dyke-Davidoff-Masson syndrome, epilepsy, imaging features

## Abstract

**Rationale:**

Dyke-Davidoff-Masson syndrome (DDMS) is a rare syndrome commonly occurring in children and characterized by cerebral hemiatrophy, hypertrophy of the skull, epilepsy, and mental retardation.^[1,2]^ However, few have been reported in China, especially in teenagers. This case investigated its possible cause and explored a relative effective solution.

**Patient concerns:**

A 24-year-old female came to department having experienced recurrent seizures for 12 years.

**Diagnosis:**

DDMS was diagnosed from its manifestations, biochemistry indexes, and imaging (computed tomography angiography, magnetic resonance venography, and so on).

**Interventions:**

Several drugs are used to treat the disease, including valproate, carbamazepine, topiramate, and ginkgo biloba extract.

**Outcomes:**

Under the medicine treatment of magnesium valproate with carbamazepine, the patient experienced partial seizures approximately once per month that lasted 30 to 60 seconds each without any complications observed during a follow-up period of 24 months.

**Conclusion:**

The imaging and clinical features of DDMS in this teenager were similar to those in classic infantile-onset cases. A potential cause of the disease could be brain trauma, which impaired the middle cerebral artery and reduced cerebral blood supply, leading to epilepsy and hemiatrophy.

**Lessons:**

It was concluded early diagnosis and pharmacotherapy are the keys to preventing intellectual decline in DDMS patients. Moreover, the combination of magnesium valproate and carbamazepine could significantly reduce the frequency and duration of seizures, despite not eliminating them completely.

## 1 Introduction

Dyke-Davidoff-Masson syndrome (DDMS) is a rare disease generally seen in children and characterized by hemiatrophy, hypertrophy of the skull, epilepsy, and mental retardation. DDMS is commonly caused by a perinatal insult,^[3,4]^ such as trauma, intrauterine infection, and hypoxic ischemic encephalopathy. Therefore, fewer cases have been reported regarding adolescents.

The case of adolescent-onset DDMS in China reveals its typical imaging features, and proposes the plausible cause of brain trauma, which impairs the middle cerebral artery (MCA) and reduces the cerebral blood supply, subsequently giving rise to epilepsy and hemiatrophy.^[5]^ In addition, cerebellar atrophy and impaired cerebellar function were also observed in this case; a relatively effective solution was explored for the disease.

## 2 Case report

A 24-year-old female was admitted to department for experiencing recurrent partial seizures for 12 years. There was no family history of DDMS. Her delivery was normal, and her only elder sister was healthy, especially the patient underwent severe craniocerebral trauma at the age of 6. She has suffered from partial seizures that attacked 2 to 3 times per month and lasted for approximately 20 seconds each from 12 years’ old. Gradually, they developed into generalized seizures that attacked 6 to 7 times per month and lasted 1 to 3 minutes each.

The patient was indifferent, only speaking a few words in a quiet voice. She was tested with MMSE, with the result of 24 point, which implied a mild intelligence decline. Her bilateral face with less facial expression was symmetry in general. Furthermore, she walked with a cerebellar ataxic gait, and it was thus difficult for her to walk with a tandem gait. Other unusual findings from her physical examination included furry legs, right facial and slight lingual paralysis, and gingival hyperplasia. Additionally, her right eyelid fissure was smaller than the left, and both eyes suffered from horizontal nystagmus. Also, the muscle strength of her right limb was measured as graded 4. Other positive signs included bilateral finger-to-nose tests, bilateral alternate motions, bilateral Heel-knee-tibia tests, and a right limb pathological sign.

**Figure 1:**
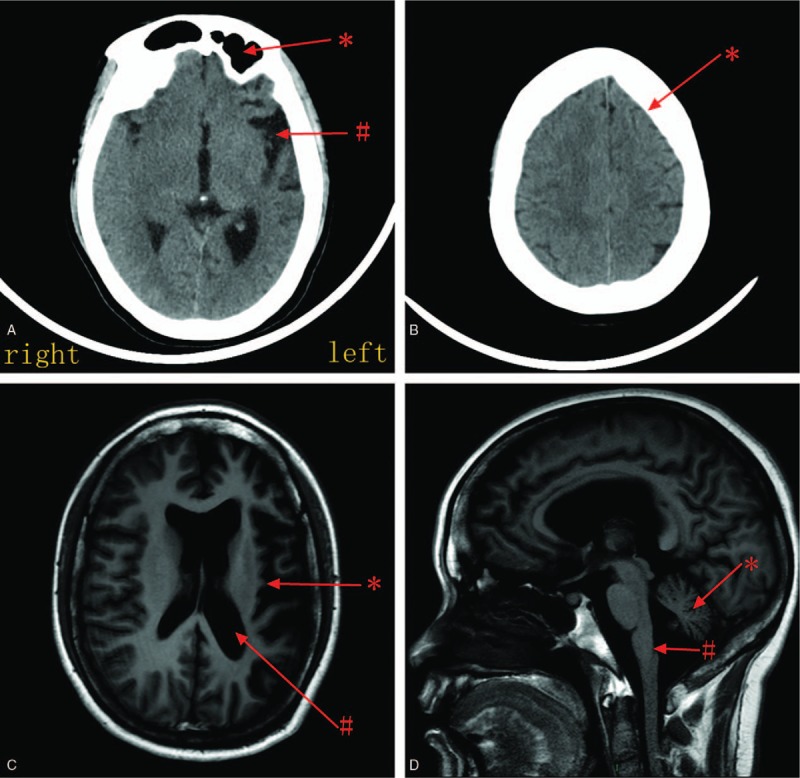
(A) Computed tomography (CT) showing atrophied left temporal lobe (#) and enlarged left frontal sinus (∗). (B) CT showing thickened left frontal skull, which turn its original arc into straight line (∗). (C) Magnetic resonance imaging (MRI) T1 showing atrophied left cerebral hemisphere (∗) and enlarged left ventricles (#). (D) MRI T1 showing atrophied cerebellum (∗) and medulla oblongata (#).

**Figure 2:**
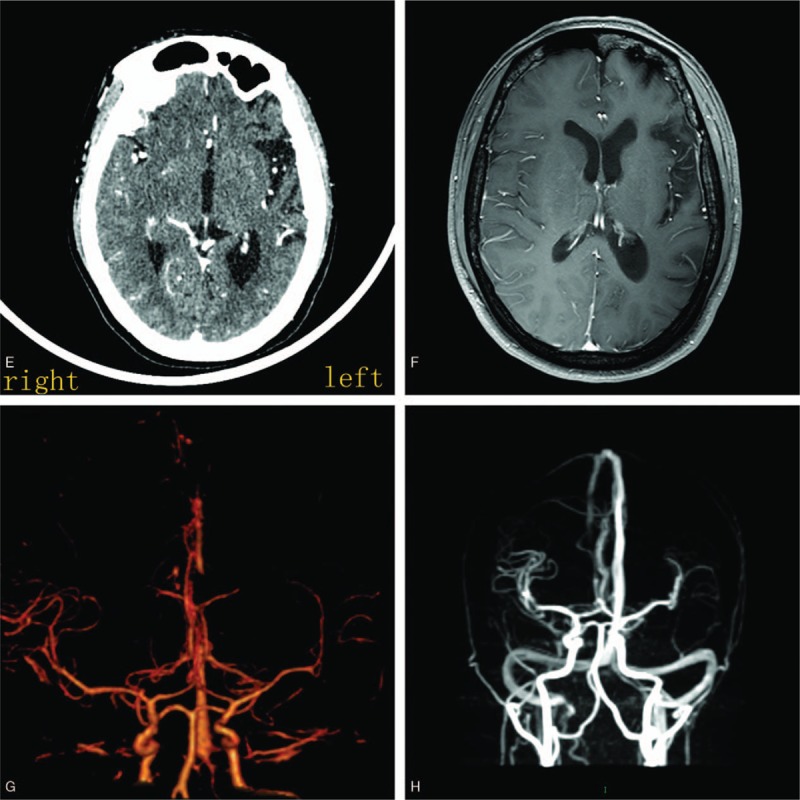
(A) Enhanced computed tomography examination showing fewer blood vessels in left cerebral hemisphere. (B) Enhanced MRI showing fewer blood vessels in left cerebral hemisphere. (C) computed tomography angiography showing fewer blood branches in left the middle cerebral artery. (D) Magnetic resonance venography showing fewer blood branches in left middle cerebral vein.

In regard to the laboratory tests, both the computed tomography and magnetic resonance imaging scans of the cranium showed the left cerebral hemisphere (Fig. 1A and C), bilateral medulla oblongata, cerebellar atrophy (Fig. 1D), and a thickened left skull (Fig. 1B). However, the left ventricles (Fig. 1C) and frontal sinus (Fig. 1A) were dilated. On the computed tomography angiography (Fig. 2A and C) and magnetic resonance venography (Fig. 2B and D), the branches of the left middle cerebral artery and vein were sparse. On cerebrospinal fluid, RBC 8 cells/mm^3^, CL^−^ 83 mmol/L↓, and other items were normal.

As DDMS is refractory epilepsy,^[6]^ following 2 years of treatment and follow-up, it was found that a combination of magnesium valproate and carbamazepine could significantly reduce the frequency and duration of the patient's seizures, but could not completely eliminate the seizures. In contrast, valproic acid combined with oxcarbazepine or topiramate did not significantly reduce the frequency nor severity of the seizure attacks. Following the superior combination treatment, the patient experienced partial seizures approximately once a month that lasts 30 to 60 seconds each. Although phenytoin sodium had some effect for the epilepsy, it was not suitable for children. And we infer that the side effect of phenytoin sodium led to gingival hyperplasia in the patient, as she had taken it for several years before coming to our department.

## 3 Discussion

There have been few reports about the onset of DDMS in adolescents.^[7]^ Specifically, in the present study, it was found that the onset of juvenile DDMS is similar to that of infantile DDMS, whose main manifestations are epilepsy, mental retardation, and so on. In addition, as there were also typical DDMS manifestations in the neuroimages, a clinical diagnosis was not difficult to obtain. Moreover, the patient suffered from cerebellar and medulla oblongata atrophy, which were less reported previously.^[8]^ Furthermore, the patient had normal intelligence before the age of 12, which was the age at which she experienced her first seizure; following this, her intelligence gradually declined, especially following the attack of 2 severe generalized seizures at the age of 19. It was inferred that this decline of intelligence as well as impairment of the balance function were both related to the brain damage caused by the recurrent epileptic seizures. Thus, it can be concluded it is essential to preserve the intelligence and balance function in DDMS patients through early diagnosis and early treatment.

Through medical image examination, it was found that the branches of the left middle cerebral artery were sparse, which could be the pathological basis of seizure for this specific case. The patient underwent severe craniocerebral trauma at the age of 6; although it did not result in a coma, her forehead region suffered from severe impact. As the result, it was inferred that this incident probably led to the impairment of the left middle cerebral artery,^[8]^ which gradually led the blood vessels to atrophy and thinning and caused the recurrent epileptic attacks eventually.

Furthermore, it was found that the patient often experienced seizure activity at night, particularly right after falling asleep. Possible reasons behind this phenomenon include the fact that cerebral blood flow velocity commonly decreases at night in normal individuals and was further affected in DDMS patient, whose branches of MCA were very sparse. As a result, the reduced cortical blood supply induced epilepsy. In addition, the vagus nerve tends to be excited at night, additionally, the “wearing-off” phenomenon of antiepileptic drugs often occurs at night, both of which contribute to nocturnal epilepsy.

## Author contributions

**Conceptualization:** Yiyang Li, Tao Zhang.

**Data curation:** Bo Li, Jing Li.

**Formal analysis:** Yiyang Li, Bo Li, Li Wang, Zhibin Jiang.

**Investigation:** Yiyang Li, Tao Zhang, Jing Li, Bo Li, Li Wang, Zhibin Jiang.

**Methodology:** Jing Li.

**Project administration:** Tao Zhang.

**Resources:** Tao Zhang, Jing Li, Li Wang.

**Writing – original draft:** Yiyang Li, Tao Zhang.

**Writing – review & editing:** Tao Zhang.
